# Development of Efficient Protocols for Stable and Transient Gene Transformation for *Wolffia Globosa* Using *Agrobacterium*

**DOI:** 10.3389/fchem.2018.00227

**Published:** 2018-06-21

**Authors:** P. P. M. Heenatigala, Jingjing Yang, Anthony Bishopp, Zuoliang Sun, Gaojie Li, Sunjeet Kumar, Shiqi Hu, Zhigang Wu, Wei Lin, Lunguang Yao, Pengfei Duan, Hongwei Hou

**Affiliations:** ^1^The State Key Laboratory of Freshwater Ecology and Biotechnology, The Key Laboratory of Aquatic Biodiversity and Conservation of Chinese Academy of Sciences, Institute of Hydrobiology, Chinese Academy of Sciences, University of Chinese Academy of Sciences, Wuhan, China; ^2^Inland Aquatic Resources and Aquaculture Division, National Aquatic Resources Research and Development Agency, Colombo, Sri Lanka; ^3^Centre for Plant Integrative Biology, University of Nottingham, Nottingham, United Kingdom; ^4^Collaborative Innovation Center of Water Security for Water Source Region of Mid-Line of South-to-North Diversion Project, College of Agricultural Engineering, Nanyang Normal University, Nanyang, China

**Keywords:** transient transformation, stable transformation, duckweed, *Wolffia globosa*, auxin, cytokinin

## Abstract

Members of the *Wolffia* genus are fascinating plants for many biologists as they are the smallest flowering plants on Earth and exhibit a reduced body plan that is of great interest to developmental biologists. There has also been recent interest in the use of these species for bioenergy or biorefining. Molecular and developmental studies have been limited in *Wolffia* species due to the high genome complexity and uncertainties regarding the stable genetic transformation. In this manuscript we present new protocols for both stable and transient genetic transformation for *Wolffia globosa* using *Agrobacterium tumefaciens*. For the transient transformation, we used *Wolffia* fronds whereas we used clusters for the stable transformation. As proof of concept we transformed two synthetic promoter constructs driving expression of the GUS marker gene, that have previously been used to monitor auxin and cytokinin output in a variety of species. Using these approaches we obtained a Transformation Efficiency (TE) of 0.14% for the stable transformation and 21.8% for the transient transformation. The efficiency of these two methods of transformation are sufficient to allow future studies to investigate gene function. This is the first report for successful stable transformation of *W. globosa*.

## Introduction

The genus *Wolffia* is a member of Lemnaceae or duckweed family. This family comprises five genera, of which members of *Wolffia* and *Wolffiella* are, the smallest angiosperms in the world (Appenroth et al., 2013). *Wolffia* plants consist of a highly reduced structure, comprising a single thallus or frond, less than 1 mm in size (Landolt, 1986; Bernard et al., 1990). *Wolffia* fronds are globular or oval shaped and the upper surface is flattened. Unlike many other members of the Lemnaceae, *Wolffia* plants are rootless. It has been reported that duckweeds absorb nutrients and water through the underside of their fronds (Leng, 1999) most likely making the root functionally redundant (Hillman, 1961; Anderdon et al., 1973). *Wolffia* increases their biomass mainly through asexual budding by producing daughter fronds within a single side pouch (basal cavity) of the mother frond (Sree et al., 2015; Ziegler et al., 2015). The process of vegetative reproduction allows *Wolffia* plants to produce genetically homogeneous populations when cultivated from a single clone and to show vigorous growth in natural environments. (Bonomo et al., 1997; Xu et al., 2011). Under favorable conditions, *Wolffia* plants are able to double their population size within 30 h (Skillicorn et al., 1993).

Under optimized growth conditions, duckweeds contain high protein with the crude protein content reaching up to 45%. Therefore, there has been increased interest in the use of *Wolffia* as a good protein source particularly for use in animal feed (Skillicorn et al., 1993; Ismail, 1998). As duckweed species have been shown to secrets certain target products into the culture medium (Firsov et al., 2015), they may be able minimize the purification cost of target proteins in duckweed based bioreactors. As many duckweeds including *Wolffia* species reproduce clonally, this allows to be grown in closed system bioreactors, which would minimize the chance of accidental release of transgenic plants (Kruse et al., 2002; Sree et al., 2015). Together with the ability of *Wolffia* species to produce genetically uniform populations from a single clone, these characteristics have made *Wolffia* easy and inexpensive to cultivate in bioreactors (Thompson, 1989). We therefore predict that research interest in duckweed species will increase within the coming years. *Wolffia* species present ideal model systems with which to study for physiological, biochemical, and genetic properties of duckweeds (Anderdon et al., 1973).

One bottleneck preventing greater use of *Wolffia* in commercial applications relates to uncertainty concerning the stable genetic transformation of *Wolffia*. Previously there have been reports of transient transformation for a number of *Wolffia* species including - *W. australiana, W. globosa*, and *W. columbiana* (Boehm et al., 2001; Kruse et al., 2002; Friedrich, 2005; Pham et al., 2010). However protocols for the stable transformation of *Wolffia* have only been reported in *W. arrihiza* (Khvatkov et al., 2015a). Estimations of genome size based on flow cytometry, have shown that *W. arrihiza* has genome size of approximately 1,881 Mbp, over 5-fold larger than the other *Wolffia* species, such as *W. australiana* (Wang et al., 2011). Therefore, there is great need to establish new protocols to allow the stable transformation of other *Wolffia* species. In this paper, we present new protocols for both the transient and stable transformation of *W. globosa*. As proof of concept we introduce to synthetic reporters (*TCS* and *DR5*) driving expression of the *GUS* reporter gene, that have previously been shown to report the cytokinin and auxin signaling output in a variety of species (Benkov et al., 2003; Müller and Sheen, 2008). Although most studies detailing novel methods of transformation use constitutive promoters as this ensures correct identification of all transformed cells, we selected to use the synthetic hormone reporters instead as they have been used successfully in our laboratory in a variety of aquatic plants, including *Hygrophila difformis* (Li et al., 2017) and *Spirodela* and *Lemna* (data unpublished). These transgenic plants will have the advantage that they will provide tools for further studies wishing to investigate growth and development in *Wolffia*, and as such, provide value beyond this study.

## Materials and methods

### Plant material and preparation of explants

*Wolffia globosa* (5563) was collected from a native population in Central China (City of Wuhan, Hubei province) at Wuhan Botanical Garden, Chinese Academy of Sciences (CAS) (30.54°N and 114.42°E). Previous work has sampled from this area confirmed the duckweed population as *Wolffia globosa* based on morphological characteristics including absence of pigment cells in the fronds, the number of stomata per frond, the size and shape of the frond and ecological adaptations and confirmed based on chloroplast mat K sequencing (Yuan et al., 2011). *W. globosa* fronds was cultured in SH medium (Schenk and Hildebrandt, 1972) and used as explants in this study. Explants were cultured under sterile conditions at 25 ± 1°C under the white light of 85 μmol m^−2^s^−1^ 16 h day/8 h night photoperiod.

### Cloning of reporter constructs

**Figure 1:**
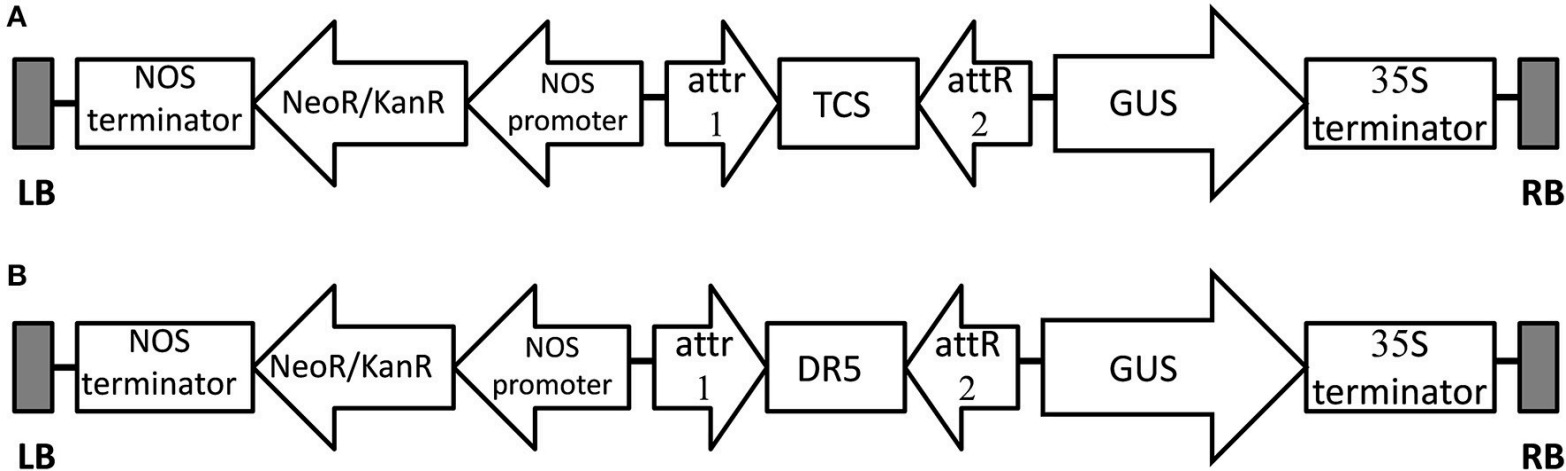
Schematic representation of T-DNA region of the expression vector TCS*::GUS*/ pKGWFS7.0 **(A)** and *DR5::GUS*/ pKGWFS7.0 **(B)**, attR & attR2 - Recombination sites for the Gateway LR reaction.

Two vector constructs were used for experiments (Figure 1). The cytokinin response element (TCS) and auxin response element (DR5) were synthesized and inserted to the binary vector pKGWFS7.0 (http://www.transgen.com.cn/) using the Gateway technology (Invitrogen). The constructs were mobilized into the commercially available disarmed *Agrobacterium* strains LBA4404 and EHA105 (http://www.transgen.com.cn/) and used for the transformation experiments.

### *Agrobacterium*-mediated transient transformation of *W. globosa*

#### Preparation of Agrobacterium

*A. tumefaciens* strains (LBA4404 and EHA105) harboring the plasmids TCS*::GUS*/pKGWFS7.0 and *DR5::GUS*/pKGWFS7.0 were cultivated in the following way. Bacteria from stock cultures were subcultured in 5 ml of Agro LB liquid medium (Table 1) supplemented with spectinomycin and rifampicin (BIOSHARP–China) and incubated at 28°C for 48 h with shaking. Cells were harvested by centrifugation at 5,000 rpm (Eppendorff 5804R, USA) for 15 min and re-suspended with 10 ml of infection medium (Table 1). Acetosyringone (AS) (Sigma-Aldrich) was only added to the infection medium after autoclaving.

**Table 1:** Composition of the media used in transient transformation.

**Media type**	**Composition**
Frond Culture Medium	SH + 2% Sucrose + 0.6% Agar
Agro LB medium	Tryptone 10 g l^−1^ + Yeast extract 5 g l^−1^ +NaCl 10 g l^−1^ + Spectinomycin 100 mg l^−1^ + Rifampicin 20 mg l^−1^, (pH - 7)
Infection Medium	Sucrose 50 g l^−1^ + Mgcl_2_(1 M) 10 ml l^−1^ + AS 10 μM l^−1^
Growth Medium	Liquid SH + 1% Sorbitol + 5% Sucrose + AS 10 μM l^−1^
Selection Medium	Liquid SH + 2% Sucrose + Cefotoxime 300 mg l^−1^ + G418 40 mg l^−1^
Frond Induction Medium	SH + 2% Sucrose + 0.6% Agar + Cefotoxime 150 mg l^−1^ +G418 40 mg l^−1^

#### Inoculation and co-cultivation of W. globosa

**Figure 2:**
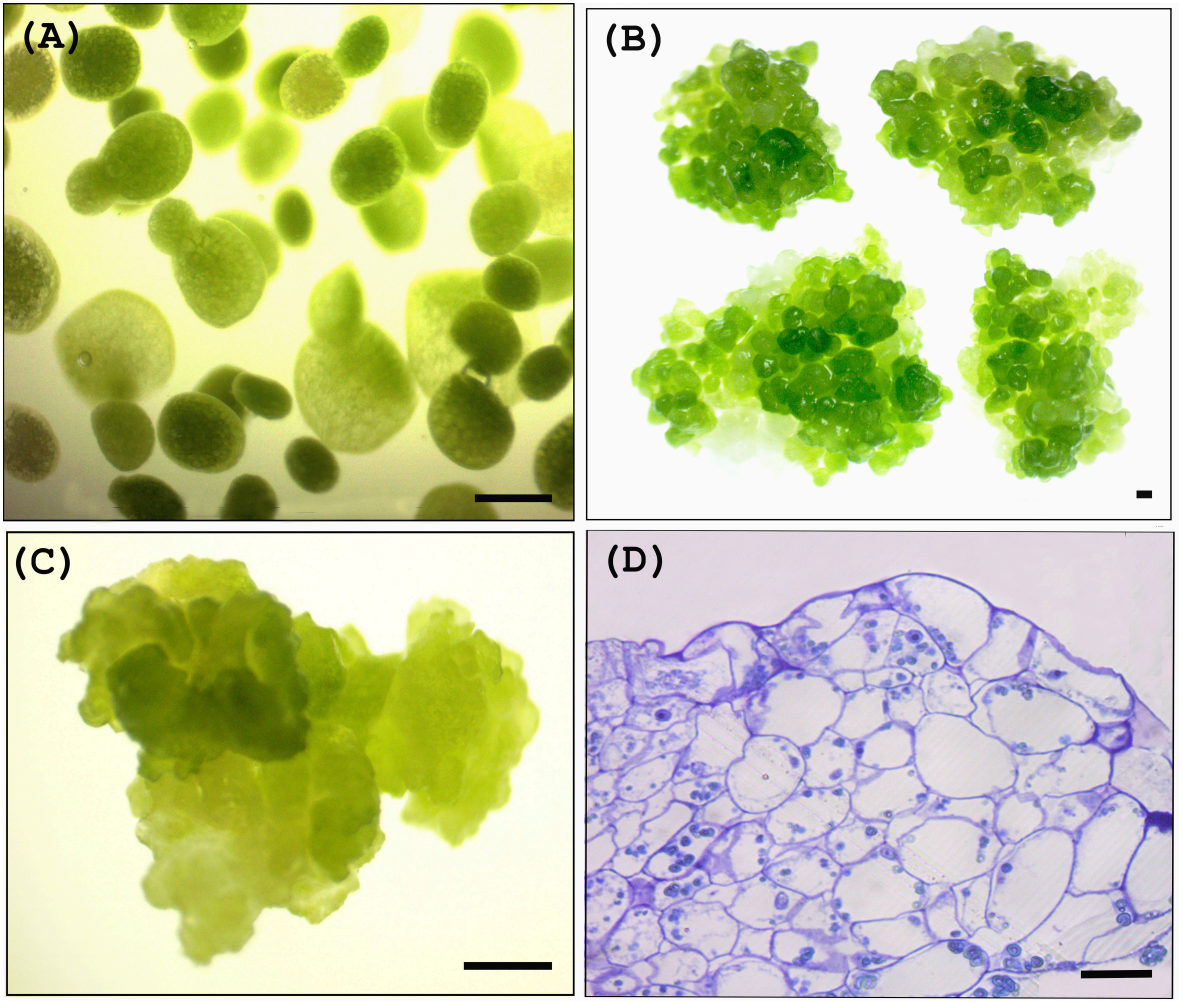
*Wolffia* plant materials used for transient and stable transformation. **(A)** *Wolffia* explants used for transient transformation, **(B)** *Wolffia* clusters used for stable transformation trials, **(C)** *Woiffia* calli used for stable transformation trials, **(D)** Cross section of calli showing cell distribution *bars*, 50 μm **(A,C)** 500 μm **(B,D)**.

Approximately 1 g of explants (Figure 2A) were placed in a 2 ml sterilized Eppendorf microcentrifuge tube containing 1 g of sterilized glass beads (1 mm). Tubes were filled with *Agrobacterium* suspended in Infection Medium and shaken at around 180 rpm for 15 min whilst maintaining the temperature at 28°C using an incubator orbital shaker (Crystal, IS-RS D3 - China). One microliter of silwet L-77 was added to the each tube after shaking. A vacuum of approximately 0.8 kg/cm^2^ was applied twice (each time for 15 min and subsequently released quickly). Explants were transferred to sterilized filter papers soaked in Growth Media (Table 1) and co-cultivated with the *Agrobacteria* for 48 h.

#### Selection of transformants

After 48 h of co-cultivation, explants were transferred into Selection Medium containing G418 and cefotaxime (BIOSHARP–China) to select resistant fronds and eliminate *Agrobacteria* (Table 1). After 1 week, resistant fronds were transferred to the Frond Induction medium (Table 1) and cultured for another week before the β-Glucuronidase (*GUS*) assay.

To identify suitable concentration for the selection of tranasgenic explants, trials were conducted with different concentrations of G418 (25, 35, 40, 50, 60, and 80 mg l^−1^) in triplicate.

#### GUS-expression assays

The histochemical assay for *GUS* activity in transgenic explants was performed according to the methodology described by Jefferson et al. (1987). G418 resistant fronds cultured in frond induction medium were immersed in Histochemical Buffer containing 0.5 mg ml^−1^ X-gluc, vacuum infiltrated (0.8 kg/cm^2^) for 30 min, and then incubated at 37°C for 12 h. After incubation, explants were washed with deionized water and 40% ethanol prior to observations under the stereomicroscope (Shunyu EX20, China). Transformation efficiency (TE) was calculated as percentage of *GUS* positive explants in the total number of explants.

### *Agrobacterium*-mediated stable transformation of *Wolffia*

#### Cluster and callus induction

Both *Wolffia* calli and clusters (fused aggregates) (Figures 2B–D) were used for *Agrobacterium* mediated stable transformation trials. Clusters and calli of *W. globosa* were induced as described by Khvatkov et al. (2015b) using the preconditioned frond explants, and maintained in SH medium. To induce clusters and calli, explants were cultured in Cluster Induction Medium for around 4 months and then transfer to Callus Induction Medium (Khvatkov et al., 2015b).

#### Transformation

*A. tumefaciens* (EHA105 containing *TCS::GUS*) was subcultured as 4 lines on solid YEB medium containing 0.8% agar, rifampicin and spectinomycin (Table 2) and grown for 48 h at 28°C. After 48 h, 4 lines were scraped and suspended in 8 ml of liquid YEB (without antibiotics). The optical density of the bacteria suspension was 0.7 ± 0.1 at 600 nm (OD_600_). Subsequently 2 g of *Wolffia* clusters were placed in falcon tube with 5 ml of bacteria suspension and 1 g of sterilized glass beads (1 mm). The tubes were subjected to 180 rpm of vigorous shaking for 30 min at 28°C in an orbital shaker. Then clusters were blot dried and co-cultivated for 72 h on filter papers soaked in Co-cultivation Medium (Table 2). Clusters were then cultured on Resting Medium (regeneration and elimination of *Agrobacteria*) (Table 2) for 2 weeks and transferred to Selection Medium (Table 2). Selection was carried out for at least 4 weeks (first 2 weeks with 2 mg l^−1^ 2,4-Dichlorophenoxyacetic acid (2,4-D) and 2 mg l^−1^ N6-benzyladenine (6-BA) and then without growth regulators for the final 2 weeks). The explants were then transferred to Frond Induction Medium and cultured for another 2 weeks.

**Table 2:** Composition of the media used in stable transformation.

**Media type**	**Composition**
YEB medium	Yeast extract 6 g l^−1^ +Tryptone 5 g l^−1^ + Sucrose 5 g l^−1^ + MgSO_4_.7H_2_O 0.5 g l^−1^ + Rifampicin 20 mg l^−1^+ Spectinomycin 100 mg l^−1^ (pH-7)
Co-cultivation Medium	Liquid SH +2% Sucrose +2 mg l^−1^ 2, 4-D + 2 mg l^−1^ 6 BA
Resting Medium	SH + 2% Sucrose +2 mg l^−1^ 2, 4-D + 2 mg l^−1^ 6 BA+ Cefotaxime 300 mg l^−1^
Selection Medium	SH + 2% Sucrose + 0.6% Agar + Cefotaxime 300 mg l^−1^+ G418 40 mg l^−1^
Frond Induction Medium	SH + 2% Sucrose + 0.6% Agar + Cefotaxime 150 mg l^−1^

All steps of both the transient and stable transformation experiments were carried out at 25 ± 1°C under the white light of 85 μmol m^−2^s^−1^, using a 16 h day/8 h night photoperiod.

#### Estimation of transformation efficiency and visualization of reporters

Transformation Efficiency (TE) was measured as described by Khvatkov et al. (2015a). After 1 month of selection, all resistant fronds from each petri dish were considered as a single transgenic population. TE was calculated as percentages of resistant fronds in the total number of explants.

#### GUS-expression assays

Histochemical assay for *GUS* activity in transgenic explants was performed according to the methodology described under the section GUS-Expression Assays.

#### Genomic analysis of transgene integration

To detect gene integration within the plant genome we firstly used a PCR assay. Total genomic DNA from the putative transgenic and wild-type *Wolffia* explants was extracted using a plant genome extraction kit, Nuclean Plant Gen DNA kit –CW BIO (http://www.cwbiotech.com.cn/). DNA extracts obtained were used as template to amplify the *TCS* element and *GUS* gene using specific primers. Primers used for TCS element was *TCS*-F: 5′-GGGACAAGTTTGTACAAAAAAGCAGGCTAGCTTTGCTAGCAAAATCTACA-3′ and *TCS*-R: 5′-GGGGACCACTTTGTACAAAAAGCTGGGTTGTTATATCTCCTTGGATCGAT-3′. Primers used for GUS gene was *GUS*-F: 5′-TCAACGGGGAAACTCAGCAAGC-3′ and *GUS*-R: 5′-CCTCCCTGCTGCGGTTTTTCA-3′.

Each PCR reaction mixture of 20 μl consisted of 2.0 μl of 10 × buffer, 0.5 μl of 10 mM dNTPs, 1 μl of reverse and forward primers each at 10 μM, 0.2 μl of Taq polymerase, 14.8 μl of deionized water and 60 ng (0.5 μl) of a DNA template. PCR was carried out for TCS element in a thermal cycler (Eppendorff, USA) at annealing temperature of 65°C and at 60°C for the *GUS* gene.

PCR products were separated on a 1.2% agarose gel and visualized under 300 nm wave length of UV. PCR products were sent for sequencing (http://www.tsingke.net) to confirm the correct sequence.

#### RT-PCR detection of the expression of gene integrated

Total RNA from wild and transformed *W. globosa* was extracted using the Trizol reagent (Invitrogen). cDNA was synthesized using 2 μg of total RNA using a Primescript RT reagent Kit (Takara). For RT-PCR the same *GUS*-specific primers (used for genomic PCR) and PCR conditions were used. An *Actin* gene was used as the internal control. As the sequence of the *Wolffia* genome is not available yet, a *Wolffia Actin* gene was first amplified using the degenerate primers. *ActF:* 5′-GTGYTKGAYTCTGGTGATGGTGT-3′ and *ActR:* 5′- ACCTTRATCTTCATGCTGCTSGG- 3′. PCR was carried out for *Actin* gene at annealing temperature of 57°C. The gene amplified was ligated to the pEASY- T1 simple vector system (Transgene) and sequenced by Tsingke company (http://www.tsingke.net). The sequence obtained was subjected to the homology search using BLAST in National Center for Biotechnology Information (NCBI, http://www.ncbi.nlm.nih.gov/) to correctly identify the gene.

### Statistical analysis

Significance of the TE parameters are shown as Standard Deviation (SD) of the mean made in triplicates and tested by *tukey*-test using SPSS software version 23.

## Results

### Transient transformation of *Wolffia globosa*

Both Transient and Stable methods of transformation have proved highly informative for many avenues of research into a myriad of molecular processes. Stable gene transformation remains the most desirable method for the long-term analysis of gene function or the long-term production of specific compounds. However in many instances, transient gene transformation may be a preferable method. In such instances transient transformation can introduce or silence genes in plants and can be used to manufacture desired protein products. Compared with stable transformation, transient transformation is often versatile, quick and efficient. We therefore set out to establish protocols for both methods of transformation in *W. globosa*.

**Figure 3:**
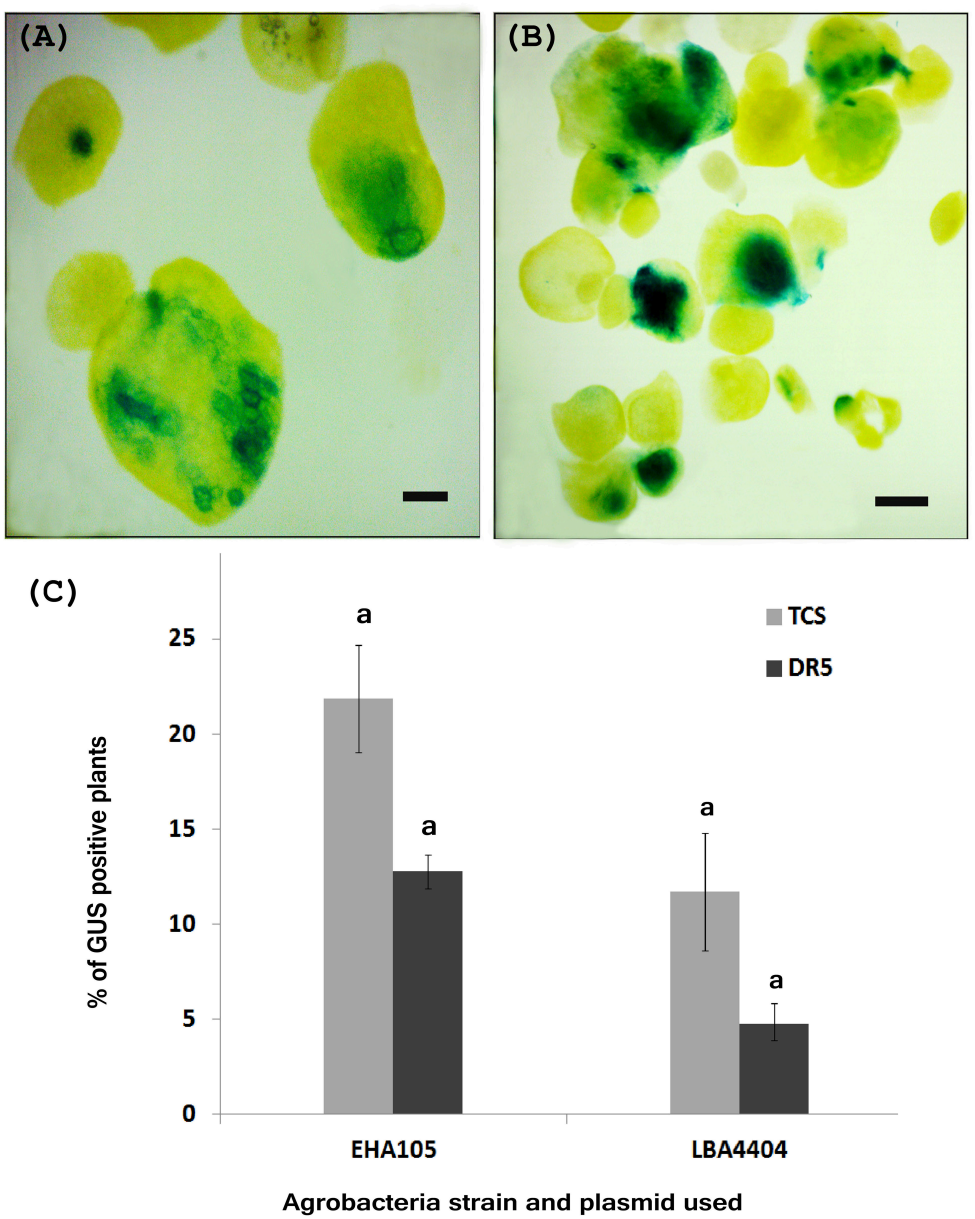
Histochemical *GUS* assay of putative transient transgenic *W. globosa* explants. **(A)** *DR5::GUS* transgenic *Wolffia*, **(B)** *TCS::GUS* transgenic *Wolffia*, **(C)** Percentage of *GUS* positive plants observed during transient transformation, with different *Agrobacteria* strains. Experiments were performed in biological triplicate. Standard deviations are indicated and letters above the bars denote significant difference between the data. *Scale bars* are 500 μm.

We developed the transient transformation protocol for *W. globosa* using *A. tumefaciens* strains EHA105 harboring either the *TCS::GUS*/pKGWFS7.0 plasmid or the *DR5::GUS*/pKGWFS7.0 plasmid. In order to optimize the transient transformation protocol, we applied a suite of different variations to the protocol. Whilst some variation in conditions was possible, the incorporation of vigorous shaking with glass beads, vacuum infiltration and incorporation of 10 μM l^−1^ AS to both the inoculation and co-cultivation media were absolute requirements successful transformation, and without these processes transformation was unsuccessful. We calculated the TE based upon the percentage of explants with positive GUS activity. Based on this we noted that transformation with *Agrobacterium* strain EHA105 gave a higher TE (21.8%) than when using the LBA4404 strain (Figure 3C). We also noted that the percentage of *TCS::GUS* transformed fronds was higher for both *Agrobacterium* strains than the number of *DR5::GUS* transformed fronds (Figure 3C).

We found that, the optimal concentration of G418 for selection of transformed lines based on antibiotic resistance was 40.0 mg l^−1^. G418 resistant explants were clearly identifiable after 2 weeks of selection. Successful transient transformation was confirmed on putative transformants using a *GUS* showing activity of the GUS transgene under control of either the TCS or DR5 promoters (Figures 3A,B).

### Stable transformation

To optimize the protocol for stable transformation of *W. globosa*, we altered several trials with different treatments and tested the efficiency of both *Agrobacterium* strains, EHA105 and LBA4404 harboring the *TCS::GUS*/pKGWFS7.0 plasmid. We only observed the stable transformation using the EHA105 strain. Additionally we observed that the clusters needed to be shaken vigorously with glass beads and the *A. tumefaciens* suspension. Trials conducted without this step were unsuccessful. Successful transformation events occurred when the optical density of *A. tumefaciens* suspension was 0.7 ± 0.1 at 600 nm (OD_600_). We also identified that the optimal co-cultivation period for the successful stable transformation of *W. globosa* with *Agrobacteria* was 3 days. After co-cultivation, subjecting plant materials for a 14 day period in Resting Medium enhanced the efficiency of transformation of the *TCS::GUS* transformed transgene. Alternative resting periods of either 5, 7, or 20 days resulted in unsuccessful transformation. After the resting period, we conducted selection in the presence and absence of growth regulators (2, 4-D and 6-BA). Omitting these growth regulators from the Selection Media resulted in the formation of no GUS positive plants.

**Figure 4:**
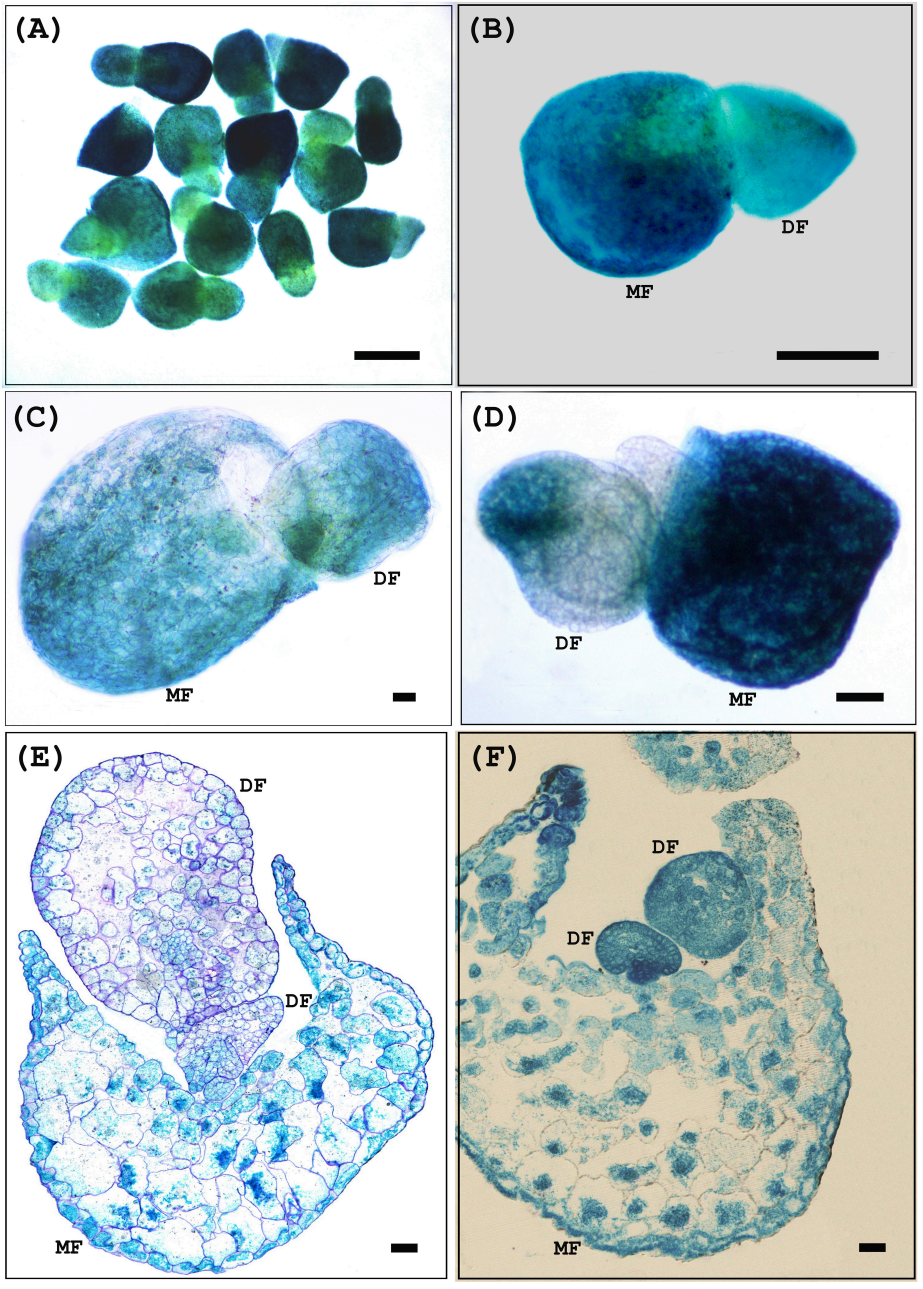
Histochemical *GUS* assay of transgenic *W. globosa* via *Agrobacterium*-mediated stable transformation after the 4 weeks of selection. **(A)** *TCS::GUS* transformed *W. globosa* transgenic fronds **(B)** *TCS::GUS* transformed *W. globosa* transgenic frond showing *GUS* expression in both mother and daughter frond. **(C,D)** *GUS* positive daughter frond comes out from the single side pouch of the stably transformed mother frond. **(E,F)** Cross sections of transgenic *W. globosa* via *Agrobacterium*-mediated stable transformation after histochemical *GUS* assay. *TCS::GUS* transformed *W. globosa* transgenic frond showing *GUS* staining in both mother and daughter frond. MF, Mother Frond; DF, Daughter Frond; Scale bars **(A–D)** - 250 μm, **(E,F)** - 50 μm.

After 4 weeks of selection, we tested G418 resistant transgenic explants for incorporation of the transgene. To confirm the incorporation and expression of the TCS::GUS transgenes, G418 resistant populations were initially analyzed for *GUS* activity and this was confirmed subsequently using molecular approaches. *GUS* staining was present in both mother and daughter fronds of transformed plants but absent from untransformed controls, confirming the stable transformation of the *TCS::GUS* gene (Figure 4).

**Figure 5:**
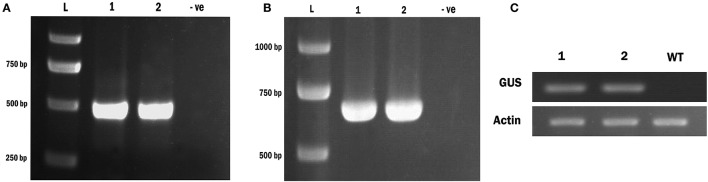
Confirmation of the e *Wolffia* transformed with *TCS::GUS* using PCR & RT- PCR analysis. **(A)** Amplification of *TCS* element from *Wolffia* transformants. **(B)** Amplification of *GUS* gene from *Wolffia* tarnsformants. For both gels a 2,000 bp ladder is used and the sizes of the corresponding bands are indicated. Lanes 1 and 2 contain two independent *TCS::GUS* transgenic plants and lane 3 (marked –ve) containes genomic DNA from a wild plant **(C)** RT-PCR for the GUS enzyme in *Wolffia* transformants. An *Actin* gene was used as internal control. Lanes 1 and 2: putative *Wolffia* transformants, WT, wild-type plant.

We also verified incorporation of the transgene using a PCR based assay. Following amplification with primers specific for either the TCS promoter element, or the GUS enzyme, we observed expected bands of 404 and 661 bp respectively in transformants (Figures 5A,B). No bands were present in our negative controls (wild-type plants). Sequencing of these fragments confirmed that they corresponded to the *TCS* and *GUS* sequences respectively.

In order to test for expression of the GUS gene using RT-PCR, we first had to identify an appropriate internal control. As the *W. globosa* genome has not been sequenced, we performed a homology search for *Actin* gene and designed appropriate *Actin* degenerate primers that showed high similarities with *Actin* gene of other plant species. We used this to identify the sequence of one of the *W. globosa Actin* genes that we could then use in subsequent assays. The sequence identified was 508 bp in length and recorded in Supplementary Data (Sequence - see Supplemental Material).

Following RNA extraction and reverse transcription, PCR products of the anticipated size (661 bp) corresponding to the *GUS* specific primers were obtained with the transgenic *Wolffia* lines but not in the control wild-type explants (Figure 5C). Therefore we conclude stable incorporation of the transgene into *W. globosa* using a number of independent assays.

The TE with *TCS::GUS* in stable transformation was 0.14 transgenic plants per 100 explants. However stable transformation with *Wolffia* callus was unsuccessful.

## Discussion

### Critical factors for successful transient transformation of *W. globosa*

During transient transformation, we found that addition of AS to the Inoculation and Co-cultivation Medium at the concentration of 10 μM l^−1^ was necessary to achieve high transformation efficiency. AS is a phenolic compound that has previously been shown to enhance T-DNA insertion into plants, therefore improving transformation efficiency (Godwin et al., 1991). This needed to be combined with vigorous shaking with glass beads and vacuum infiltration, and presumably these treatments aid the passage of *Agrobacteria* in to the plant cells. Our results are consistent with those of other researchers, for example Boehm et al. (2001), reported unsuccessful transient transformation with *W. columbiana* when either particle or vacuum treatments were not used prior to infection.

We noted that the percentage of *GUS* positive plants was higher in transient transformation conducted using *Agrobacteria* strain EHA105 compared with to using LBA4404 strain (Figure 3C) and therefore propose that the *Agrobacteria* strain EHA105 is more suitable for the transformation of *W. globosa*.

### Critical factors for successful stable transformation of *W. globosa*

In this study we found the following factors were critical to obtaining high transformation efficiencies. The concentration of *A. tumefaciens* suspension must have an optical density of 0.7 ± 0.1 at 600 nm (OD_600_). Other researchers have previously shown that bacterial cell density is an important factor greatly affecting for the TE (Yang et al., 2010; Chhabra et al., 2011). However, there is some variation in what level is optimal, for example, Khvatkov et al. (2015a) reported successful stable transformation of *Wolffia arrhiza* with the cell density of 0.4–0.6 at 600 nm (OD_600_). Several factors, including plant species, type of explants, *A. tumefaciens* strain vector type and the infection and co-cultivation conditions may collectively affect the gene transformation (Hiei et al., 2000). Therefore, we would expect different outcomes for different *Wolffia* species under different, experimental conditions.

In our stable transformation assays, the *Agrobacterium* strains EHA 105 worked efficiently, and based upon this observation we propose that the EHA105 is more suitable for *W. globosa* transformation than the LBA 4404 strain.

In our experiments we found it necessary that the plant material to be transformed were damaged by shaking with glass beads to improve the accessibility of the infecting bacteria. Several studies have confirmed that wounding plant materials (using either microprojectile bombardment, shaking with glass beads, scratching or slicing) prior to co-cultivation often increased the TE (Grayburn and Vick, 1995; Boehm et al., 2001; Hoshi et al., 2004). Wounding of plant cells can enhance attraction of *Agrobacterium* to the wound site by releasing AS, which also induces the transformation of *A. tumefaciens* virulence genes (Usami et al., 1987). However, Khvatkov et al. (2015a) has reported successful stable transformation of *W. arrhiza* without this treatment and whilst we find that it is needed for *W. globosa*, we note that this may not be the case for other plant species or under different experimental conditions.

Co-cultivation period also a critical factor that affects for the successful transformation and shorter or longer co-cultivation periods lead to unsuccessful transformation (Aileni et al., 2011). In our study, we identified 3 days as the optimal duration for co-cultivation of *W. globosa* and *Agrobacterium*. Longer co-cultivation periods (4–5 days) caused over proliferation of *Agrobacterium* around the plant materials ultimately leading to death of the plant tissues.

After the co–cultivation, plant materials were kept in Resting Medium. This step is essential to all of material to recover from the co-cultivation shock. We found 14 days of resting time before selection to be the optimal conditions for stable transformation in *W. globosa*. This is in accordance with the findings of Khvatkov et al. (2015a). To eliminate *Agrobacteria*, we initially used cefotaxime at a high concentration (300 mg l^−1^), but we reduced this in subsequent weeks, and eventually found this could be eliminated. Applying cefotaxime in liquid medium was more effective, presumably because the whole surface of the plant was in contact with the antibiotics solution. We therefore found it best to culture plants on filter papers soaked in liquid Resting Medium for the first week after co-cultivation to aid in the successful removal of *Agrobacteria*.

We attempted stable transformation with both clusters and calli of *Wolffia* however we only achieved successful transformation using clusters. This results was similar to the findings of Khvatkov et al. (2015a) and according to their report, transformation of *Wolffia* with calli was inefficient due to calli undergoing necrosis (Khvatkov et al., 2015a).

## Conclusions

We report here new protocols for both *Agrobacterium* mediated transient and stable genetic transformation systems for *W. globosa*. Using these protocols, transgenic *Wolffia* plants can be produced within 18 days by transient transformation and stable lines can be obtained in 65 days starting with *Wolffia* clusters. The development of these new protocols for transformation open paths to utilize this valuable plant species for a wide range of scientific and commercial processes.

## Author contributions

HH planned and designed the research. PH, JY, ZS, GL, SK, SH, ZW, WL, LY, and PD performed experiments and data analysis. PH, AB, HH, SK, and ZW wrote the manuscript. All authors contributed to manuscript revision, read and approved the submitted version.

### Conflict of interest statement

The authors declare that the research was conducted in the absence of any commercial or financial relationships that could be construed as a potential conflict of interest.
