# Supplementary material for: Development of Efficient Protocols for Stable and Transient Gene Transformation for Wolffia Globosa Using Agrobacterium
Source: Front Chem. 2018 Jun 21;6:227. doi: 10.3389/fchem.2018.00227 (PMC6022245; doi:10.3389/fchem.2018.00227)
Supplement: Supplementary file 1 [file Data_Sheet_1.PDF]

frontiers

*Supplementary Material*

# Development of efficient stable and transient gene transformation protocols for *Wolffia globosa* using *Agrobacterium*.

**P. P. M. Heenatigala, Jingjing Yang, Zuoliang Sun, Gaojie Li, Sunjeet Kumar, Shiqi Hu, Zhigang Wu, Wei Lin, Lunguang Yao, Pengfei Duan, Hongwei Hou***

*** Correspondence:** Corresponding Author: houhw@ihb.ac.cn

## 1 Supplementary Data

Sequence of *Wolffia Actin* gene used for RT-PCR

**5'-**
GTGTTGGATTCTGGTGATGGTGTAACACACACAGTGCCGATCTACGAAGGCTATGCACT
TCCCCACGCAATCCTAAGGCTGGATCTCGCTGGAAGGGATCTGACCGACGCTCTGATGA
AGATTTTGACAGAGAGGGGCTACTCCTTCACCACCACAGCCGAACGGGAAATTGTGAGG
GACATCAAGGAGAAGCTGGCCTACGTGGCACTAGACTACGAGCAGGAGCTGGAGACGG
CCAAATCAAGCTCGTCTGTGGAGAAGACCTATGAGCTGCCCGACGGGCAGGTGATCACC
ATCGGGGCCGAGAGATTCCGCTGCCCGGAGATCCTGTTCCAGCCCTCGTTGGTGGGAAT
GGAGGCGGCGGGCATCCACGAGACGACATACAACTCGATCATGAAGTGCGATGTGGAC
ATCAGGAAGGATCTGTACGGGAACATCGTGCTCAGTGGAGGGACAACGATGTTCCCGGG
CATCGCGGACCGCATGAGCAAGGAGATCACTGCGCTTG**- 3'**
