# Supplementary material for: Development of Efficient Protocols for Stable and Transient Gene Transformation for Wolffia Globosa Using Agrobacterium
Source: Front Chem. 2018 Jun 21;6:227. doi: 10.3389/fchem.2018.00227 (PMC6022245; doi:10.3389/fchem.2018.00227)
Supplement: Supplementary file 3 [file Image_2.PDF]

Supplementary Material

Development of efficient stable and transient gene transformation protocols for *Wolffia globosa* using *Agrobacterium*.

P. P. M. Heenatigala, Jingjing Yang, Zuoliang Sun, Gaojie Li, Sunjeet Kumar, Shiqi Hu, Zhigang Wu, Wei Lin, Lunguang Yao, Pengfei Duan, Hongwei Hou*

*** Correspondence:** Corresponding Author: houhw@ihb.ac.cn

1 Supplementary Image 2

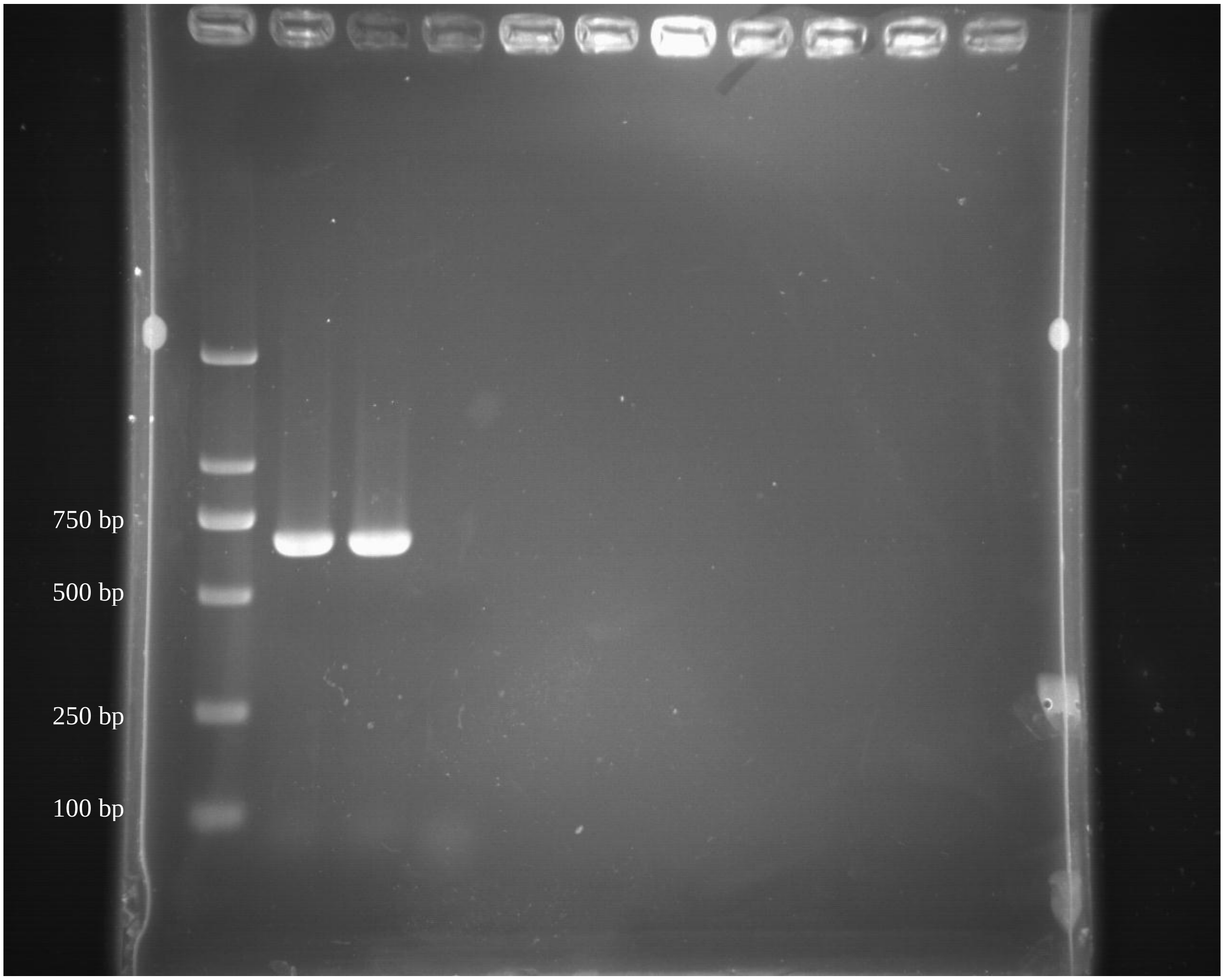

Supplementary Image 2. Original gel photograph - Confirmation of *GUS* gene (Expected band size 661bp) integration into the *Wolffia* genome by PCR. (**Lane1** – 2000 bp ladder, **Lane 2 &3**: *TCS::GUS* transgenic plants; **Lane 4**: Negative control, genomic DNA of wild plant was used as template)
