# Supplementary material for: Development of Efficient Protocols for Stable and Transient Gene Transformation for Wolffia Globosa Using Agrobacterium
Source: Front Chem. 2018 Jun 21;6:227. doi: 10.3389/fchem.2018.00227 (PMC6022245; doi:10.3389/fchem.2018.00227)
Supplement: Supplementary file 4 [file Image_3.PDF]

Supplementary Material

Development of efficient stable and transient gene transformation protocols for *Wolffia globosa* using *Agrobacterium*.

P. P. M. Heenatigala, Jingjing Yang, Zuoliang Sun, Gaojie Li, Sunjeet Kumar, Shiqi Hu, Zhigang Wu, Wei Lin, Lunguang Yao, Pengfei Duan, Hongwei Hou*

*** Correspondence:** Corresponding Author: houhw@ihb.ac.cn

1 Supplementary Image 3

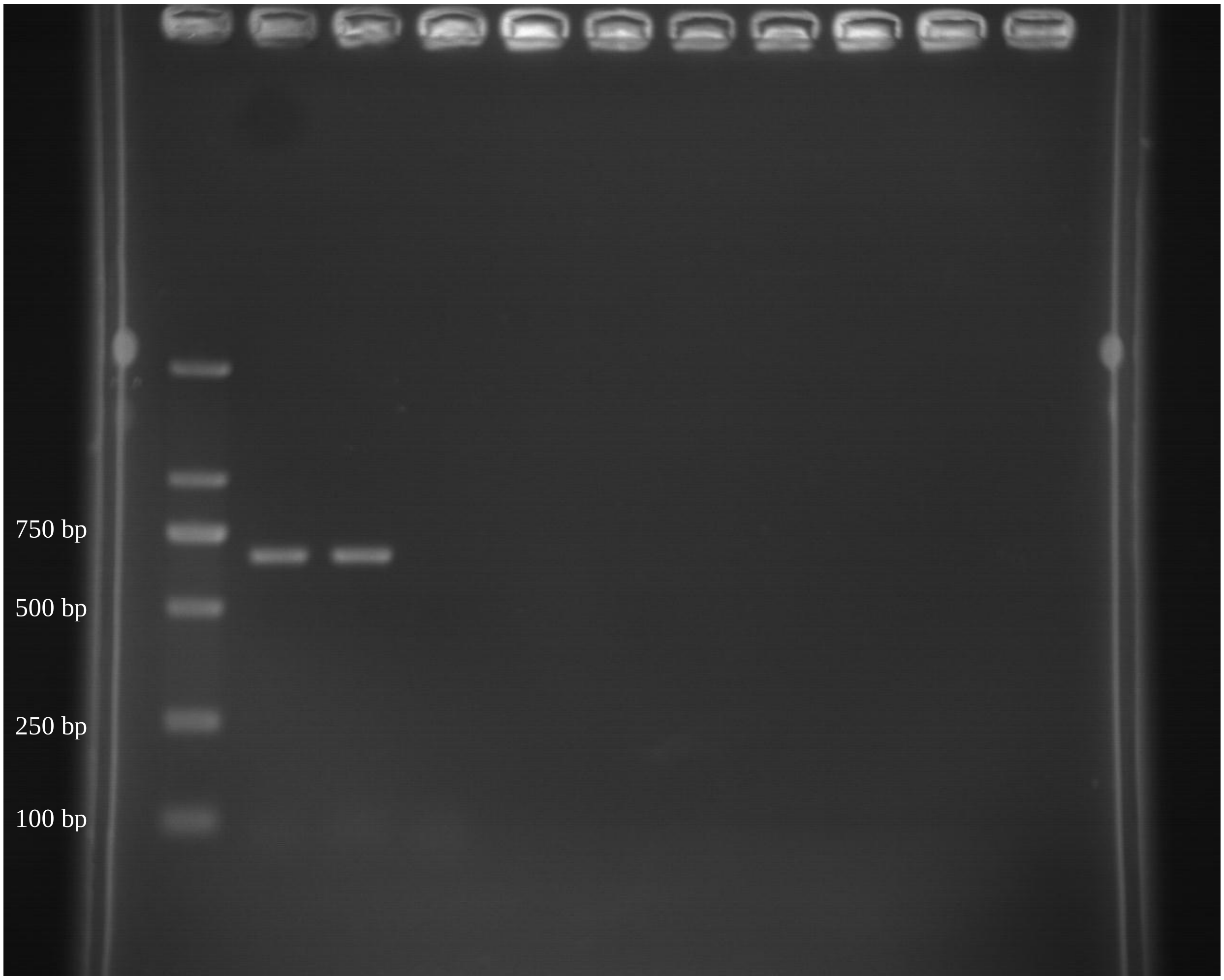

Supplementary Image 3. Original gel photograph - RT-PCR analysis for expression of *GUS* gene (expected band soze 661 bp) in *Wolffia*. **Lane1**: 2000 bp ladder, **Lanes 2& 3**: putative *Wolffia* transformants **, Lanes 4:**Negative control wild type *wolffia*
